# Turner syndrome: fertility, familial clustering, and cancer risk

**DOI:** 10.1093/humrep/deae266

**Published:** 2024-12-10

**Authors:** K Allen-Brady, L E Verrilli, B A Austin, J M Letourneau, E B Johnstone, C K Welt

**Affiliations:** Division of Epidemiology, Department of Internal Medicine, University of Utah School of Medicine, Salt Lake City, UT, USA; Division of Reproductive Endocrinology and Infertility, Department of Obstetrics and Gynecology, University of Utah School of Medicine, Salt Lake City, UT, USA; Intermountain Health, Department of Obstetrics and Gynecology, Murray, UT, USA; Division of Endocrinology, Metabolism and Diabetes, Department of Internal Medicine, University of Utah School of Medicine, Salt Lake City, UT, USA; Division of Reproductive Endocrinology and Infertility, Department of Obstetrics and Gynecology, University of Utah School of Medicine, Salt Lake City, UT, USA; Division of Reproductive Endocrinology and Infertility, Department of Obstetrics and Gynecology, University of Utah School of Medicine, Salt Lake City, UT, USA; Division of Endocrinology, Metabolism and Diabetes, Department of Internal Medicine, University of Utah School of Medicine, Salt Lake City, UT, USA

**Keywords:** primary ovarian insufficiency, pregnancy, colon cancer, prostate cancer, familial clustering, mosaic Turner syndrome, oocyte donation

## Abstract

**STUDY QUESTION:**

Is there an increased risk of reproductive or colon cancer in women with Turner syndrome and their family members?

**SUMMARY ANSWER:**

Our data suggest that there is an increased risk for sigmoid colon cancer in women with Turner syndrome and an increased prostate cancer risk in second- and third-degree male relatives.

**WHAT IS KNOWN ALREADY:**

Turner syndrome has been associated with lower risk of breast cancer, but increased risk of gonadoblastoma and colon cancer in some, but not all studies. There is also evidence for a genetic predisposition to sex chromosome aneuploidy, which may indicate a predisposition to Turner syndrome and the associated cancer risk in family members.

**STUDY DESIGN, SIZE, DURATION:**

The study was a retrospective case–control study of women with Turner syndrome diagnosed from 1995 to 2021, their relatives, and population subjects from Utah.

**PARTICIPANTS/MATERIALS, SETTING, METHODS:**

Women with Turner syndrome were identified using International Classification of Disease (ICD) codes in electronic medical records from two major Utah healthcare systems and reviewed for accuracy. Women with Turner syndrome were linked to genealogy in the Utah Population Database. Cancer diagnoses (breast, ovarian, endometrial, colon, testicular, and prostate) were determined for women with Turner syndrome and their relatives using the Utah Cancer Registry. Live births to women with Turner syndrome were identified by linked birth certificates. The relative risk of cancer in women with Turner syndrome and in relatives was estimated by comparison to population rates matched by age, sex, and birthplace.

**MAIN RESULTS AND THE ROLE OF CHANCE:**

We identified 289 women with Turner syndrome. Sigmoid colon cancer was increased in women with Turner syndrome (OR [95% CI] 24.2 [2.9, 87.4]; *P* = 0.0032). There were 233 women with Turner syndrome who had at least three generations of genealogical data. There was an increased risk of Turner syndrome in first- (OR [95% CI] 18.08 [2.19, 65.32]; *P *=* *0.0057) and second-degree relatives (9.62 [1.17, 34.74]; *P *=* *0.019), although numbers were very small. There was an increased risk of prostate cancer in second- (1.8 [1.4, 2.2]; *P* < 0.001) and third-degree relatives (1.3 [1.1, 1.5]; *P* < 0.001). There was no increased risk of colon cancer in relatives.

**LIMITATIONS, REASONS FOR CAUTION:**

Based on the small number of sigmoid colon cancer cases, it is possible that our data have overestimated the colon cancer risk. Limitations include the identification of Turner syndrome by a diagnosis code in one of the two major health systems in Utah. The population is largely northern European with 9.3% of the women self-identified as Hispanic and 2.4% as Native American or multiple races. The results may not be generalizable to other populations.

**WIDER IMPLICATIONS OF THE FINDINGS:**

Our data suggest that women with Turner syndrome may need early screening for colon cancer. Additional studies are needed to identify risk factors for sex chromosome aneuploidy and cancer risk in women with Turner syndrome and their male relatives.

**STUDY FUNDING/COMPETING INTEREST(S):**

The work in this publication was supported by R56HD090159 and R01HD099487 (C.K.W.). We also acknowledge partial support for the Utah Population Database through grant P30 CA2014 from the National Cancer Institute. The Utah Cancer Registry is funded by the National Cancer Institute’s SEER Program, Contract No. HHSN261201800016I, the US Centers for Disease Control and Prevention’s National Program of Cancer Registries, Cooperative Agreement No. NU58DP007131, with additional support from the University of Utah and Huntsman Cancer Foundation. The content is solely the responsibility of the authors and does not necessarily represent the official views of the National Institutes of Health. The authors have no conflicts of interest.

**TRIAL REGISTRATION NUMBER:**

N/A.

## Introduction

Turner syndrome is classically diagnosed with a 45,X karyotype or partial loss of the X chromosome along with characteristic features (Gravholt *et al.*, 2023). However, the wide range of X chromosome aneuploidies and complicated karyotypes make the phenotypes quite heterogeneous. Sex chromosome abnormalities include undiagnosed mosaic Turner karyotypes, which may not manifest typical Turner syndrome risks (Berglund *et al.*, 2020; Tuke *et al.*, 2019). Therefore, large datasets are important to understand Turner syndrome fertility and cancer risk.

Turner syndrome is not typically thought to be heritable. However, rare, deleterious variants in *USP26* are increased in fathers of patients with Klinefelter’s syndrome (Liu *et al.*, 2021). The study suggests that there could be a hereditary component to sex chromosome aneuploidy in families.

Previous studies examining cancer risk in women with Turner syndrome have been performed in large cytogenetic registries and centers in Sweden, Denmark, and Great Britain (Gravholt *et al.*, 1998; Schoemaker *et al.*, 2008a; Ji *et al.*, 2016; Viuff *et al.*, 2021). Risk of some types of cancers appear to be increased but results have been conflicting and additional data are needed (Gravholt *et al.*, 1998; Schoemaker *et al.*, 2008a; Ji *et al.*, 2016; Viuff *et al.*, 2021).

Using the Utah Population Database (UPDB), a population-based genealogy from the state of Utah linked to electronic medical records (EMRs), cancer records, and birth certificates, we examined familial clustering, and reproductive and colon cancer risks in women with Turner syndrome. We were also able to examine cancer risk in the first-, second-, and third-degree relatives of these women, which has not been done previously, to our knowledge. Our data support and expand previous fertility and cancer findings in population registries and suggest a heritable component to Turner syndrome and cancer risk.

## Materials and methods

### Turner cases

We identified women with Turner syndrome using EMRs from 1995 to 2021 at the University of Utah Health and Intermountain Healthcare, which together serve 85% of all residents in Utah. Cases of Turner syndrome were ascertained through International Classification of Disease (ICD) codes: ICD-9 (758.6) and ICD-10 codes (Q96). The initial list of identified Turner cases was verified through individual chart review of adult records by a medical or reproductive endocrinologist (C.K.W. or L.E.V.) for inclusion. We recorded the karyotype when it was available in the adult records (Table 1). We excluded Turner cases with XY karyotype assigned male at birth. Additional subjects were identified in a search for cases of women with primary ovarian insufficiency, in which chart review indicated a diagnosis of Turner syndrome as the cause of primary ovarian insufficiency (POI) (Verrilli *et al.*, 2023). We estimated a population prevalence of Turner syndrome using the population of women in Utah from the 2022 United States Census, 1.619 million, multiplied by 0.85 as the approximate percentage of the Utah population who seek care in the two health systems queried. Age at diagnosis was documented as birth year subtracted from diagnosis year. However, in cases for which the diagnosis year was clearly older than the presumed diagnosis date, the diagnosis age was taken as 14 years, an age at which the patient should have presented with delayed puberty.

**Table 1.** Characteristics of Turner Syndrome subjects.

Karyotype (n)	Turner (n = 175)	45,X (n = 56)	Mosaic 45,X/46,XX (n = 32)	Mosaic 45,X/46,XXX (n = 8)	Mosaic 45,X/46,XY (n = 7)	Other (n = 11)	All (n = 289)
Diagnosis age (years)	10.6 ± 7.4^a^	5.4 ± 10.0^b^	10.7 ± 8.1^a,b^	6.2 ± 5.3^a,b^	4.4 ± 6.4^a,b^	8.2 ± 5.6^a,b^	9.2 ± 8.2
Current age (years)	35.9 ± 16.2^a^	23.0 ± 13.1^b^	36.5 ± 19.4^a^	19.4 ± 8.9^b^	30.1 ± 15.7^a,b^	20.3 ± 6.9^b^	32.9 ± 6.6
Height (cm)	148.9 ± 7.8	151.0 ± 6.0	150.1 ± 7.7	144.2 ± 3.2	154.4 ± 3.7	152.8 ± 5.8	149.7 ± 7.3
BMI (kg/m^2^)	28.1 ± 8.1	29.7 ± 9.6	31.1 ± 8.3	25.2 ± 5.3	27.5 ± 8.0	26.7 ± 4.6	28.6 ± 8.2
Growth hormone use*	17.7	64.3	15.6	62.5	42.9	54.5	29.8
Estradiol or hormonal contraception	40.6	73.2	50.0	25.0	71.4	54.5	48.8
Smoker	3.4	3.6	12.5	0	0	0	4.1
Alcohol use	5.7	1.8	6.2	0	14.3	0	4.8
Hypothyroid	24.6	30.4	25.0	0	14.3	9.1	24.2
Celiac sprue	3.4	7.1	3.1	25.0	0	9.1	4.8
Crohn’s disease	1.1	0	0	0	0	0	0.7
Ulcerative colitis	0.6	0	6.2	0	0	0	1.0
Type 2 diabetes	9.7	0	15.6	0	0	0	7.6
Type 1 diabetes	1.7	0	0	0	0	0	1.0

Available karyotype descriptions are presented, with the designation Turner used for subjects that did not have an available karyotype in the adult medical record. Diagnosis age, current age, height, and BMI are presented as mean ± SD. Other data are presented as percentages within a group. Diagnosis age (ANOVA *P *=* *2.6 × 10^−4^) and current age (ANOVA *P *=* *2.2 × 10^−7^) were different among karyotype categories, with differences by *post hoc* testing indicated with different letters. There was no difference in height or BMI between the groups. There was a difference in the proportion of women treated with growth hormone in the different karyotype categories (*Chi-square *P *=* *2.8 × 10^−7^). There was no difference in the proportion of women who used estradiol or hormonal contraception, smoked, drank alcohol or had hypothyroidism, celiac sprue, inflammatory bowel diseases, or type 1 or type 2 diabetes as a function of karyotype categories.

### Pedigree creation

We used the Utah Population Database (UPDB) to link genealogy information to medical record information and data from the Utah Cancer Registry (UCR). Medical record numbers for women with Turner syndrome were converted to UPDB identification numbers by an independent oversight group under the requirements for use. Subsequently, UPDB IDs were linked to genealogy contained within the UPDB. For the familiality analysis, Turner probands were required to have at least three generations of genealogy information available (proband, both parents, and one set of grandparents). Three generations increase the likelihood that complete family data, health care, and UCR data are available for any woman within the UPDB.

### Ethics

The University of Utah and Intermountain Healthcare Institutional Review Boards and the Resource for Genetic and Epidemiologic Research (RGE), overseers of UPDB data, approved this study.

### Assessment of familiality

Familiality is the familial clustering of disease in significant excess compared to matched population rates of disease. To assess familial clustering, we estimated the relative risk (RR) of Turner syndrome in first-, second-, and third-degree relatives. RR was estimated as the ratio of the observed number of cases of Turner syndrome for a specific relative type (e.g. first-degree relatives) compared to the expected number of Turner syndrome cases for the specific relative type. The expected number of affected female relatives was calculated based on population rates of Turner syndrome for each 5-year female birth cohort represented by the Turner syndrome cases and birth place (Utah or outside of Utah) within the University of Utah Health and Intermountain Healthcare.

### Demographic and associated data

The current age of women with Turner syndrome and their relatives was calculated as 2024-birth year, unless there was a death date for the individual. If the individual was deceased, death age was taken as the current age. BMI and height for Turner subjects were obtained from the most recent visit in the EMR or from driver’s licenses in subjects over 18 years of age to ensure that growth was complete. ICD codes for hypothyroidism (ICD-9 244.X, ICD-10 E03.X and E06.X), type 1 diabetes mellitus (250.X, E10.X), celiac disease (579.0, K90.0), Crohn’s disease (555.X, K50.X), and ulcerative colitis (556.X, K51.X) were obtained from the EMR. Children born to women with Turner syndrome were determined using birth certificate data available through 2020.

### Identification of cancer cases

Probands with a verified Turner diagnosis and their family members were screened for cancer diagnoses using the existing linkage between the UPDB and the UCR. UCR securely transmitted these variables to the UPDB for linkage to other study variables and for release to the investigator.

### Cancer relative risk analysis

We estimated the RR of individual cancers in women with Turner and their first-, second-, and third-degree relatives. Of note, first-degree relatives include parents, siblings, and children of cases. Second-degree relatives include grandparents, aunts/uncles, nieces/nephews, half-siblings, and grandchildren. Third-degree relatives include great grandparents, great grandchildren, and first cousins. The RR of a disease estimates how likely an individual is to develop a disease if she has Turner syndrome or is a relative of a woman with Turner syndrome. RR was estimated as the ratio of the observed number of cases of a specific type of cancer for women with Turner syndrome or a specific relative type (e.g. first-degree relatives) compared to the expected number of cancer cases for women with Turner syndrome or their specific relative type. The expected number of cancer cases was calculated based on population rates, calculated for each 5-year birth cohort represented by the Turner syndrome cases or relatives, sex, and birthplace (Utah or outside of Utah) within the University of Utah Health and Intermountain Healthcare. The rate was defined by the total number of cancer cases within a cohort divided by the total cohort size. The number of expected cases was calculated as the sum of each cohort-specific cancer rate for each Turner syndrome case or their relatives of a specific type (e.g. first-degree relatives). Approximate 95% confidence intervals and exact hypothesis tests of the null hypothesis (RR = 1.0) were constructed assuming that the number of cancer cases found among the relatives follows a Poisson distribution. We corrected for multiple testing using Bonferroni correction for four cancer types in women (*P* < 0.05/4 = 0.012) and three cancer types in men (*P* < 0.05/3 = 0.017).

Many RR sampling schemes can lead to bias or inflated estimates. The studies we performed with the UPDB are population-based, reducing the risk for sampling bias that results from proband identification and over-sampling from pedigrees with multiple affected members.

## Results

We identified 289 women with confirmed Turner syndrome (Table 1). The estimated prevalence based on this number is 2.1 per 10 000 women in the state of Utah. Their ages were 9.1 ± 8.2 years (median age 12 years, range: birth-67 years) at diagnosis. The subjects diagnosed at older ages (over 50 years) were diagnosed as 45,X after presenting with a X-linked recessive disease, choroideremia, or with primary ovarian insufficiency after age 30 years rather than at the time of puberty (mosaic karyotype). The current age of the subjects was 40.0 ± 16.4 years (median age 29 years, range 7–74 years). The karyotype was not available for the majority of subjects (n = 175). The rest were documented 45,X, mosaic 45,X/46,XX, 45,X/47,XXX, 45,X/46,XY, and other (Table 1). There was a difference in diagnosis age and current age among the different karyotype categories and a difference in growth hormone use, but BMI, and the proportion with inflammatory bowel diseases, celiac sprue, and diabetes were not different (Table 1). Overall, Crohn’s disease, ulcerative colitis, type 1 diabetes, celiac disease, and hypothyroidism identified using ICD codes were increased in women with Turner syndrome compared to population rates (Table 2).

**Table 2.** Associated autoimmune diseases in women with Turner Syndrome (n = 289).

Disease	Observed #	Expected #	Relative risk (95% CI)	*P*-value
Crohn’s disease	≤10	0.32	9.26 (1.91, 27.05)	0.0045
Ulcerative colitis	≤10	0.34	8.93 (1.84, 26.10)	0.0049
Type 1 diabetes	11	1.03	10.73 (5.36, 19.20)	1.3 × 10^−8^
Celiac disease	12	0.62	19.46 (10.06–34.00)	3.6 × 10^−12^
Hypothyroidism	84	4.50	18.69 (14.90–23.13)	<0.001

The observed number of subjects with associated disease, expected number based on population rates, and the relative risk. The relative risk of these autoimmune diseases was increased in women with Turner syndrome. Observed numbers were listed as ≤10 based on the regulations of the UPDB oversight board, the Resource for Genetic and Epidemiologic Research, to protect the identity of small numbers of patients. Bonferroni multiple testing correction *P *<* *0.01.

There were 233 women with Turner syndrome who had three generations of family members present in the UPDB. Of those relatives who were still alive, first-degree relatives had a median age of 42 years with 70% ≤60 years, second-degree relatives had a median age of 48 years with 50% ≤60 years, and third-degree relatives had a median age of 37 years with 42% ≤60 years. A larger percentage of the third-degree relatives were deceased (40.1%) compared to first-degree relatives (11.9%) or second-degree relatives (26.4%).

There was an increased RR of having Turner syndrome in first- and second-degree relatives of women with Turner syndrome, although the numbers were very small. The RR of Turner syndrome in first-degree relatives was increased (OR [95% CI] 18.08 [2.19, 65.32]; *P *=* *0.0057) and based on one set of concordant, monozygotic twins with Turner syndrome confirmed by karyotype. The RR in second-degree relatives was also increased (9.62 [1.17, 34.74]; *P *=* *0.019) based on a single pair. There was no increased risk in third-degree relatives (3.20 [0.39, 11.55]; *P *=* *0.13).

We examined children born to women with Turner syndrome. Some women with Turner syndrome did have children (<5%). Sixty percent of these achieved unassisted pregnancies and were aged 20–30 years at the time of their 1–2 births and had mosaic karyotypes. The other 40% had one child through oocyte donation between ages 25 and 30 years.

There was a nominal increase in colon cancers in women with Turner syndrome and a significant increase in sigmoid colon cancer compared to population rates (Table 3, Bonferroni corrected *P *<* *0.0125). Sigmoid colon cancer cases were diagnosed between ages 45 and 55 years. The women with sigmoid colon cancer did not have a history of smoking or alcohol use, celiac sprue, or inflammatory bowel disease. In women with Turner syndrome and sigmoid colon cancer, there was no documented growth hormone use and the women had negative genetic testing for genes associated with Lynch syndrome.

**Table 3.** Cancer identified in women with Turner Syndrome (n = 289).

Cancer type	Observed #	Expected #	Relative risk (95% CI)	*P*-value
Breast	≤10	1.94	1.03 (0.12, 3.73)	0.72
Ovarian	≤10	0.22	4.58 (0.12, 25.54)	0.20
Uterine	≤10	0.37	5.34 (0.95, 19.28)	0.055
Colon	≤10	0.21	9.39 (1.14, 33.91)	0.020
Sigmoid colon	≤10	0.08	24.19 (2.93, 87.38)	0.0032^*^

The observed number of subjects with cancer, expected number based on population rates, and the relative risk. The relative risk of sigmoid colon cancer is increased in women with Turner syndrome. Observed numbers were listed as ≤10 based on the regulations of the UPDB oversight board, the Resource for Genetic and Epidemiologic Research, to protect the identity of small numbers of patients. Bonferroni multiple testing correction

* *P *<* *0.012.

The nominally increased risk in colon cancer and significant increase in sigmoid colon cancer remained when considering only the smaller number of women with Turner syndrome who had 3 generations of family data available in the UPDB (Table 4, *P *<* *0.0125). Prostate cancer risk was higher in second- and third-degree relatives of women with Turner syndrome. There was a nominally increased risk of colon and sigmoid cancer in third-degree relatives. There was no increased risk of Crohn’s disease, ulcerative colitis, type 1 diabetes, or hypothyroidism in relatives.

**Table 4.** Cancer in women with Turner Syndrome and their first-, second-, and third-degree relatives.

Relative type	Median age (quartile 1, quartile 3) (years)	Cancer type	Observed	Expected	Relative risk (95% CI)	*P*-value
Self (n = 233)		Breast	≤10	1.69	1.18 (0.14, 4.28)	0.69
		Ovarian	≤10	0.20	4.89 (0.12, 27.27)	0.18
		Uterine	≤10	0.35	2.90 (0.07, 16.14)	0.29
		Colon	≤10	0.19	10.44 (1.26, 37.70)	0.016
		Sigmoid	≤10	0.07	27.59 (3.34, 99.65)	0.0025^*^
First degree males (n = 489) females (n = 484)	42 (24, 57)	Breast	13	8.75	1.49 (0.79, 2.54)	0.17
		Ovarian	≤10	0.99	0.00 (0.00, 3.71)	1.00
		Uterine	≤10	1.81	0.55 (0.01, 3.08)	1.00
		Colorectal	≤10	2.51	1.20 (0.25, 3.49)	0.74
		Sigmoid	≤10	0.90	1.11 (0.03, 6.19)	0.59
		Prostate	11	7.85	1.40 (0.70, 2.51)	0.28
		Testicular	≤10	0.81	2.47 (0.30, 8.92)	0.19
Second degree (n = 1390 male, n = 1343 female)	48 (34, 65)	Breast	47	38.44	1.22 (0.90, 1.63)	0.17
		Ovarian	≤10	4.61	1.09 (0.35, 2.53)	0.81
		Uterine	≤10	8.60	0.81 (0.33, 1.68)	0.73
		Colorectal	13	13.66	0.95 (0.51, 1.63)	1.00
		Sigmoid	≤10	4.67	0.64 (0.13, 1.88)	0.64
		Prostate	71	40.53	1.75 (1.37, 2.21)	0.000013^*^
		Testicular	≤10	2.31	0.43 (0.01, 2.41)	0.74
Third degree (n = 4258 male, n = 4185 female)	37 (20, 64)	Breast	127	115.44	1.10 (0.92, 1.31)	0.28
		Ovarian	11	14.86	0.74 (0.37, 1.32)	0.43
		Uterine	27	28.44	0.95 (0.63, 1.38)	0.93
		Colorectal	65	49.29	1.32 (1.02, 1.68)	0.032
		Sigmoid	26	16.69	1.56 (1.02, 2.28)	0.036
		Prostate	172	132.39	1.30 (1.11, 1.51)	0.00094^*^
		Testicular	≤10	4.63	0.43 (0.05, 1.56)	0.34

The relative type and number, observed number of subjects with cancer, expected number based on population rates, and the relative risk. The relative risk of sigmoid colon cancer is increased in women with Turner syndrome and prostate cancer was increased in male second- and third-degree relatives. Observed numbers were listed as ≤10 based on the regulations of the UPDB oversight board, the Resource for Genetic and Epidemiologic Research, to protect the identity of small numbers of patients. Bonferroni multiple testing correction

* *P *<* *0.012 for women and

* *P *<* *0.017 for men.

## Discussion

We report familial clustering, pregnancies, and cancer risk in women with Turner syndrome through a population study in Utah. The data suggest that while rare, there were two cases, each, of Turner syndrome shared among first- and second-degree relatives. The data also support previous studies suggesting that it is mainly women with mosaic Turner syndrome that have children. Finally, we demonstrate an increased risk for sigmoid colon cancer in women with Turner syndrome and prostate cancer in second- and third-degree male relatives.

Although there are rare reports of mother-to-daughter transmission of Turner syndrome, which would be the most likely mode of first-degree relative sharing, our study found no transmission of Turner syndrome from mothers to daughters. The first-degree relatives we did identify were concordant, monozygotic 45,X twins, who would therefore have a different mechanism for sharing their karyotype. Monozygotic twins with Turner syndrome have been reported rarely (Shine and Corney, 1966; Riekhof *et al.*, 1972). Most twin cases are mosaic, occurring after the formation of the zygote. Therefore, discordance is seen among many monozygotic twin births with only one of the twins exhibiting Turner syndrome (Riekhof *et al.*, 1972), unlike the current case.

Recent studies suggest that sex chromosome aneuploidy may be inherited (Liu *et al.*, 2021). The study demonstrated that variants in *USP26* are associated with fathering of children with Klinefelter syndrome, suggesting that there could be a genetic cause for fathering children with sex chromosome aneuploidies (Liu *et al.*, 2021). In light of the data, we examined familial clustering and found an increased RR of Turner syndrome in second-degree relatives. While the RR is statistically increased, the absolute risk of Turner syndrome among relatives is extremely small.

Variability in ovarian function in women with Turner syndrome has been explained by mosaicism. It was previously demonstrated that some women with mosaic Turner syndrome had a normal reproductive lifespan and pregnancy rates that are not different from those of women with a 46,XX karyotype, although live births were not examined in this study (Tuke *et al.*, 2019). Based on case reports above, it is rare for women with a 45,X karyotype to have a live birth (Tuke *et al.*, 2019). In our population-based study with births documented by birth certificates, we found that only subjects with Turner syndrome caused by mosaic karyotypes had a live birth. The small number of subjects using donor oocyte may reflect the overall decreased motherhood in women with Turner syndrome, which includes decreased marriage rates (Stochholm *et al.*, 2012). There may also be limitations on access to donor oocyte programs for these women.

We identified an increased risk of sigmoid colon cancer and a nominally increased risk of colon cancer in women with Turner syndrome. We did not retrieve cases of rectal cancer but did find a case on chart review. Colon cancer has previously been described in women with Turner syndrome, but epidemiologic studies are inconsistent (Gravholt *et al.*, 1998; Schoemaker *et al.*, 2008a; Ji *et al.*, 2016; Viuff *et al.*, 2021). The largest study performed within the Danish Health registry demonstrated an increased risk of colon cancer (Gravholt *et al.*, 1998; Viuff *et al.*, 2021). Our data provide additional support. Celiac and inflammatory bowel disease were found in Turner syndrome at increased rates compared to the Utah population and increase colon cancer risk, although the women diagnosed with colon cancer had not been diagnosed with these disorders. The women in our study developed colon cancer between ages 45 and 55 years, emphasizing the relatively early onset colon cancer in these women. The current study would therefore support early screening for colon cancer in women with Turner syndrome.

Growth hormone treatment is standard of care in Turner syndrome to improve short stature (Gravholt *et al.*, 2023). While there is no data that follows growth hormone risks into old age, the largest study does not suggest increased colon cancer risk from growth hormone treatment for non-cancer related use (Swerdlow *et al.*, 2017). There was no documented growth hormone use in the women with colon cancer in our study.

We did not examine other types of cancer that appear to be increased in Turner syndrome, including meningiomas, melanoma, and bladder cancers (Gravholt *et al.*, 1998; Schoemaker *et al.*, 2008a; Ji *et al.*, 2016; Viuff *et al.*, 2021). We found one possible case of gonadoblastoma, although pertinent records were not available to confirm. Of note, women with Turner syndrome have a decreased risk of breast cancer (Gravholt *et al.*, 1998; Schoemaker *et al.*, 2008a; Ji *et al.*, 2016; Viuff *et al.*, 2021). We found very few cases, but the RR was not different than for the matched population. Rather than comparing expected breast cancer by calculating cancer rates (Schoemaker *et al.*, 2008a; Ji *et al.*, 2016), we used actual cancer cases in the population (Viuff *et al.*, 2021). Further, Utah has a relatively low breast cancer rate compared to the other United States (https://gis.cdc.gov/Cancer/USCS/#/StatePrevalence/, accessed 8/2024). We did not analyze risk based on hormone replacement but note that the only cases of reproductive cancers were not associated with hormone use beyond the recommended age (Gravholt *et al.*, 2023). These factors, along with small numbers, may have resulted in failure to identify lower breast cancer risk in women with Turner syndrome in Utah.

We found that second- and third-degree relatives of women with Turner syndrome had an increased risk of prostate cancer. The increased risk was not present in first-degree relatives, possibly related to their young age with only 30% >60 years. In contrast, 50% second-degree and 58% third-degree relatives were >60 years. The link between Turner syndrome and prostate cancer risk in families could have multiple explanations. A family history of heritable cancers, such as colon cancer, has been associated with increased risk of prostate cancer. Lynch syndrome has a risk of both colon and prostate cancer (Ryan *et al.*, 2014). However, in our study, the observed colon cancer cases had been genetically tested for Lynch syndrome and were negative. Other potential cancer risk genes could lie on the X chromosome (Xu *et al.*, 1998). Environmental factors, such as pesticides or smoking, could also be involved as it is possible that these families live in similar regions across the state or have similar lifestyles. However, we do not have the information available in the current study.

The strengths of the study are the identification of Turner syndrome cases at a population level that have been validated with chart review, the extensive genealogy database to identify relatives, and the UCR to confirm cancer in the population. Limitations of our study include the inability to identify Turner cases that were not given a diagnosis code in one of the two major health systems in Utah. Nevertheless, our population prevalence is similar to those previously reported based on population-based studies in Northern Europe (Schoemaker *et al.*, 2008b; Ji *et al.*, 2016; Berglund *et al.*, 2019). We did not have karyotypes available on all subjects and were unable to evaluate cancer risk as a function of the karyotype, which would be of interest. We also have no information about pregnancy terminations related to a Turner karyotype, which limits the interpretation of heritability of both cancer and Turner syndrome in families. However, Utah has the fifth lowest rate of pregnancy termination in the USA (Rate of Legal Abortions per 1000 Women Aged 15–44 Years by State of Occurrence | KFF), suggesting that terminations related to Turner syndrome are also likely very low. The population in Utah is largely northern European, but 9.3% of the women with Turner syndrome self-identified as Hispanic and 2.4% as Native American or multiple races. However, our data may not be generalizable to other races and ethnicities.

Our data examined comorbidities in women with Turner syndrome diagnoses across the state of Utah based on phenotype and with a diagnostic code in the EMR. The data suggest that there is limited familial clustering based on rare cases of Turner syndrome shared by first- and second-degree relatives. The women with Turner syndrome who had a live birth had a mosaic karyotype. We also found an increased risk of sigmoid colon cancer in women with Turner syndrome and a higher rate of prostate cancer in second- and third-degree relatives. These data should contribute to risk discussion and early screening and preventive strategies for women with Turner syndrome.

## Data Availability

The data underlying this article cannot be shared publicly due to regulations of the UPDB oversight board, the Resource for Genetic and Epidemiologic Research. The data will be shared on reasonable request to the corresponding author.

## References

[deae266-B2] Berglund A , Stochholm K, Gravholt CH. The epidemiology of sex chromosome abnormalities. Am J Med Genet C Semin Med Genet 2020;184:202–215. 32506765 10.1002/ajmg.c.31805

[deae266-B3] Berglund A , Viuff MH, Skakkebæk A, Chang S, Stochholm K, Gravholt CH. Changes in the cohort composition of turner syndrome and severe non-diagnosis of Klinefelter, 47,XXX and 47,XYY syndrome: a nationwide cohort study. Orphanet J Rare Dis 2019;14:16. 30642344 10.1186/s13023-018-0976-2 PMC6332849

[deae266-B4] Bernard V , Donadille B, Zenaty D, Courtillot C, Salenave S, Brac de la Perriere A, Albarel F, Fevre A, Kerlan V, Brue T et al; CMERC Center for Rare Disease. Spontaneous fertility and pregnancy outcomes amongst 480 women with Turner syndrome. Hum Reprod 2016;31:782–788. 26874361 10.1093/humrep/dew012

[deae266-B7] Gravholt CH , Juul S, Naeraa RW, Hansen J. Morbidity in Turner syndrome. J Clin Epidemiol 1998;51:147–158. 9474075 10.1016/s0895-4356(97)00237-0

[deae266-B8] Gravholt CH , Viuff M, Just J, Sandahl K, Brun S, van der Velden J, Andersen NH, Skakkebaek A. The changing face of Turner syndrome. Endocr Rev 2023;44:33–69. 35695701 10.1210/endrev/bnac016

[deae266-B10] Ji J , Zoller B, Sundquist J, Sundquist K. Risk of solid tumors and hematological malignancy in persons with Turner and Klinefelter syndromes: a national cohort study. Int J Cancer 2016;139:754–758. 27061708 10.1002/ijc.30126

[deae266-B11] Liu C , Liu H, Zhang H, Wang L, Li M, Cai F, Wang X, Wang L, Zhang R, Yang S et al Paternal USP26 mutations raise Klinefelter syndrome risk in the offspring of mice and humans. EMBO J 2021;40:e106864. 33978233 10.15252/embj.2020106864 PMC8246067

[deae266-B15] Riekhof PL , Horton WA, Harris DJ, Schimke RN. Monozygotic twins with the Turner syndrome. Am J Obstet Gynecol 1972;112:59–61. 5007509 10.1016/0002-9378(72)90530-3

[deae266-B16] Ryan S , Jenkins MA, Win AK. Risk of prostate cancer in Lynch syndrome: a systematic review and meta-analysis. Cancer Epidemiol Biomarkers Prev 2014;23:437–449. 24425144 10.1158/1055-9965.EPI-13-1165

[deae266-B17] Schoemaker MJ , Swerdlow AJ, Higgins CD, Wright AF, Jacobs PA; UK Clinical Cytogenetics Group. Cancer incidence in women with Turner syndrome in Great Britain: a national cohort study. Lancet Oncol 2008a;9:239–246. 18282803 10.1016/S1470-2045(08)70033-0

[deae266-B18] Schoemaker MJ , Swerdlow AJ, Higgins CD, Wright AF, Jacobs PA; United Kingdom Clinical Cytogenetics Group. Mortality in women with turner syndrome in Great Britain: a national cohort study. J Clin Endocrinol Metab 2008b;93:4735–4742. 18812477 10.1210/jc.2008-1049

[deae266-B19] Shine IB , Corney G. Turner's syndrome in monozygotic twins. J Med Genet 1966;3:124–128. 5963205 10.1136/jmg.3.2.124 PMC1012914

[deae266-B20] Stochholm K , Hjerrild B, Mortensen KH, Juul S, Frydenberg M, Gravholt CH. Socioeconomic parameters and mortality in Turner syndrome. Eur J Endocrinol 2012;166:1013–1019. 22436401 10.1530/EJE-11-1066

[deae266-B21] Swerdlow AJ , Cooke R, Beckers D, Borgstrom B, Butler G, Carel JC, Cianfarani S, Clayton P, Coste J, Deodati A et al Cancer risks in patients treated with growth hormone in childhood: the SAGhE European Cohort Study. J Clin Endocrinol Metab 2017;102:1661–1672. 28187225 10.1210/jc.2016-2046 PMC6061931

[deae266-B22] Tuke MA , Ruth KS, Wood AR, Beaumont RN, Tyrrell J, Jones SE, Yaghootkar H, Turner CLS, Donohoe ME, Brooke AM et al Mosaic Turner syndrome shows reduced penetrance in an adult population study. Genet Med 2019;21:877–886. 30181606 10.1038/s41436-018-0271-6 PMC6752315

[deae266-B23] Verrilli L , Johnstone E, Welt CK, Allen-Brady K. Primary ovarian insufficiency has strong familiality: results of a multigenerational genealogical study. Fertil Steril 2023;119:128–134. 36283864 10.1016/j.fertnstert.2022.09.027 PMC10024920

[deae266-B24] Viuff MH , Stochholm K, Lin A, Berglund A, Juul S, Gravholt CH. Cancer occurrence in Turner syndrome and the effect of sex hormone substitution therapy. Eur J Endocrinol 2021;184:79–88. 33112259 10.1530/EJE-20-0702

[deae266-B25] Xu J , Meyers D, Freije D, Isaacs S, Wiley K, Nusskern D, Ewing C, Wilkens E, Bujnovszky P, Bova GS et al Evidence for a prostate cancer susceptibility locus on the X chromosome. Nat Genet 1998;20:175–179. 9771711 10.1038/2477

